# Serum Metabolic Profiling of Late-Pregnant Women With Antenatal Depressive Symptoms

**DOI:** 10.3389/fpsyt.2021.679451

**Published:** 2021-07-08

**Authors:** Qiang Mao, Tian Tian, Jing Chen, Xunyi Guo, Xueli Zhang, Tao Zou

**Affiliations:** ^1^Shanghai Key Laboratory of Forensic Medicine (Academy of Forensic Science), Shanghai, China; ^2^Department of Psychiatry, The Affiliated Hospital of Guizhou Medical University, Guiyang, China; ^3^Department of Pharmacology, The Second Affiliated Hospital of Chongqing Medical University, Chongqing, China; ^4^Department of Neurology, The Affiliated Hospital of Guizhou Medical University, Guiyang, China; ^5^Department of Neurology, Yongchuan Hospital of Chongqing Medical University, Chongqing, China; ^6^Department of Psychiatry, Linyi Mental Health Center, Linyi, China

**Keywords:** antenatal depression, metabolomics, edinburgh postnatal depression scale, biomarker, amino acid metabolism, glycerophospholipid metabolism

## Abstract

**Background:** Antenatal depression (AD) is a major public health issue worldwide and lacks objective laboratory-based tests to support its diagnosis. Recently, small metabolic molecules have been found to play a vital role in interpreting the pathogenesis of AD. Thus, non-target metabolomics was conducted in serum.

**Methods:** Liquid chromatography—tandem mass spectrometry—based metabolomics platforms were used to conduct serum metabolic profiling of AD and non-antenatal depression (NAD). Orthogonal partial least squares discriminant analysis, the non-parametric Mann–Whitney *U* test, and Benjamini–Hochberg correction were used to identify the differential metabolites between AD and NAD groups; Spearman's correlation between the key differential metabolites and Edinburgh Postnatal Depression Scale (EPDS) and the stepwise logistic regression analysis was used to identify potential biomarkers.

**Results:** In total, 79 significant differential metabolites between AD and NAD were identified. These metabolites mainly influence amino acid metabolism and glycerophospholipid metabolism. Then, PC (16:0/16:0) and betaine were significantly positively correlated with EPDS. The simplified biomarker panel consisting of these three metabolites [betaine, PC (16:0/16:0) and succinic acid] has excellent diagnostic performance (95% confidence interval = 0.911–1.000, specificity = 95%, sensitivity = 85%) in discriminating AD and NAD.

**Conclusion:** The results suggested that betaine, PC (16:0/16:0), and succinic acid were potential biomarker panels, which significantly correlated with depression; and it could make for developing an objective method in future to diagnose AD.

## Introduction

Antenatal depression (AD), defined as depression during pregnancy up to the point of birth, is one of the most widespread psychiatric disorders and produces harmful effect on the mother and the infant's health (1, 2). Systematic reviews determined that the prevalence of antenatal had a significant increasing trend in the last decade and varied in different countries and regions, such as 19.7% in mainland China, 50% in Nepal, and 30–40% in South Africa (3–5). Women with AD are more likely to develop several complications, including an increased risk of nausea, vomiting, miscarriage, and poor cognitive and fetal growth, which even develop into postpartum depression (6). For offspring, AD not only results in prematurity to the fetus or in low birth weight, but also has a steady adverse effect on their brain, behavior, immune function, and hypothalamic–pituitary–adrenal (HPA) function (3, 7, 8). Therefore, AD is a still major public health issue worldwide, and it is very important to find better measures to prevent and treat AD.

As it is well-known, depression disorder is greatly influenced by genetics and environmental factors, as well as their interactions (9, 10). AD is accompanied by physiological and psychological changes, which results in alteration of hormones, inflammatory cytokines, HPA axis (11–14). Christian et al. have reported that depressive symptoms of AD are associated with elevated serum pro-inflammatory cytokines [interleukin 6 (IL-6) and tumor necrosis factor α] among pregnant women (15). Similarly, higher cerebrospinal fluid IL-1β, IL-23, and IL-33 concentrations were significantly associated with increased odds of perinatal depression (16). However, there are still no empirical laboratory methods to diagnose antepartum depression. Currently, AD diagnosis heavily depends on clinical features and syndrome, identified by experienced psychiatrists (17). As a result, many patients could be misdiagnosed or never diagnosed for the high heterogeneity of depressive symptoms (17–19). Thus, an effective diagnostic method for AD would be of considerable clinical significance.

Metabolomics, a promising tool in non-invasive biomarkers for diagnosis, has been widely used to identify global or targeted endogenous metabolites by detecting various biosamples, such as urine and plasma (20, 21). Currently, liquid chromatography–mass spectrometry (LC-MS), nuclear magnetic resonance spectroscopy, and gas chromatography–MS are the three main analytical techniques of metabolomics and are suitable for non-targeted metabonomic mapping (22).However, LC-MS was generally employed in both metabolomic and proteomic research to identify novel biomarkers for depression, characterized by high sensitivity, high resolution, and wide coverage of metabolites and peptides (23–25). There are a few studies of plasma metabolomics in AD to screen and identify some biomarkers (26, 27). Wu et al. have found that PC (18:2(2E,4E)/0:0) and cholesterol sulfate were increased in the plasma of AD and significantly associated with AD (26). Mitro et al. (27) reported that triacylglycerol metabolites and betaine were related with the incidence of antepartum depression; C48:5 triacylglycerol and C50:6 triacylglycerol were related with higher odds of AD, whereas betaine was associated with lower percentage of AD. However, no study has yet explored changes to metabolomes in late-pregnancy women with depression symptoms.

Thus, a serum metabonomic method in the diagnosis of AD was evaluated. LC-MS–based serum metabolomic platforms were conducted to distinguish metabolic profiling of AD and non-antenatal depression (NAD). Besides, orthogonal partial least squares discriminant analysis was used to identify the differential metabolites between the two groups; Spearman's correlation between key differential metabolites and Edinburgh Postnatal Depression Scale (EPDS), and the stepwise logistic regression analysis was used to identify potential biomarkers.

## Materials and Methods

### Subject Recruitment

The protocols of this research were reviewed and approved by the Medical Ethics Committee of The Affiliated Hospital of Guizhou Medical University. All participants were asked to sign informed consent before sample collection; also, all procedures were conducted according to the principles expressed in the Declaration of Helsinki. In present study, 60 subjects were recruited from the psychiatry department of the Affiliated Hospital of Guizhou Medical University from September 2015 to September 2016. All singleton-pregnancy subjects were screened by licensed obstetricians and psychiatrists. The inclusion criteria were as follows: (1) late pregnancy (gestational weeks ≥28); (2) parity ≤ 4; (3) between the ages of 22 and 40 years; (4) body mass index (BMI) at prepregnancy >18.5 and <30 kg/m^2^; (5) complete clinical data; and (6) no history of mental or cognitive illness before pregnancy. The exclusion criteria were as follows: (1) educational level <9 years; (2) history of psychoactive substance abuse; (3) with severe systemic diseases such as metabolic encephalopathy and liver or kidney disease; and (4) complications associated with pregnancy.

The diagnosis of perinatal depression (AD) was implemented based on a renowned approach as described in the previous studies (17, 26). All subjects need to fill out the assessment scale, named EPDS, and EPDS is a self-administered instrument that includes 10 statements, scored from 0 to 3, rendering a maximum score of 30. Subjects with an EPDS-10 ≥10 were defined as AD subjects, whereas the NAD group subjects with EPDS-10 <10 (17).

### Sample Collection and Preparation

The fasting blood was collected from the elbow vein of subjects in the morning using 5-mL Vacutainer tubes. Then, the blood was allowed to static 30 min at room temperature and followed by centrifugation at 3,000 revolutions/min (rpm) for 15 min. Each aliquot (150 μL) of the plasma sample was stored at −80°C until further analyses. The sample extraction protocol was based on previously published literature (28). Briefly, 50-μL sample and 150 μL of extract solution [acetonitrile: methanol = 50:50 (vol/vol), containing isotopically-labeled internal standard mixture], were taken to an Eppendorf tube (1.5 mL), vortexed the samples for 30 s, and sonicated for 15 min. Then, these were centrifuged at 12,000 rpm for 15 min at 4°C followed by incubation for 60 min at −40°C (29). Finally, the supernatant was collected and transferred to a new sample vial for LC-MS/MS analysis. Quality control samples were prepared by pooling the equivalent supernatants of all samples.

### LC-MS/MS Analysis

The Vanquish™ UHPLC system (Thermo Fisher Scientific, Waltham, MA, USA) with a Waters ACQUITY UPLC BEH Amide column (2.1 × 100 mm, 1.7 μm) was used to separate the metabolites, respectively. The temperature of column and autosampler was set at 25°C and 4°C, respectively, and the volume of injection was 3 μL The mobile phase (A) was prepared by dissolving 25 mmol/L ammonium acetate and ammonia hydroxide in water and adjusted the pH of solution to 9.75, and the mobile phase (B) consisted of 100% acetonitrile. The analysis was carried out with elution gradient as follows: 0–0.5 min, 5% A; 0.5–7.0 min, 5–35% B; 7.0–8.0 min,35–60% A; 8.0–9.0 min, 60% A; 9.0–9.1 min, 60–5% A; 9.1–12.0 min, 5% A (30).

The Q Exactive HFX mass spectrometer (Orbitrap MS, Thermo Fisher Scientific) system and MS/MS data acquisition control were performed by acquisition software (Xcalibur™ 4.0 software, Thermo). Specification of the ESI source was set as follows (30): (1) the flow rate of sheath gas and Aux gas flow rate were 50 and 10 Arb; (2) the capillary temperature was 320°C; (3) full MS resolution and MS/MS resolution of 60,000 and 7,500, respectively; (4) collision energy of 10/30/60 in NCE mode; and (5) spray voltage of 3.5 kV (positive) or −3.2 kV (negative), respectively.

### Statistical and Bioinformatics Analysis

The raw data from metabolites were converted to the mzML format using Proteo Wizard MS converter and processed with an in-house program, which was developed using R and based on XCMS, for peak detection, extraction, alignment, and integration. Internal standard normalization was employed to linearly shift the RT across the entire run for metabolite analysis (31). Then, peak annotation was processed by an in-house MS/MS database. Subsequently, the data matrix was imported into SIMCA-P V16.0.2 software (Umetrics, Umea, Sweden) for multivariable statistical analysis. Then, the orthogonal projection to latent structures–discriminant analyses (OPLS-DA) model was applied to visualize the discrimination between the AD and NAD groups in both positive and negative models. The variable importance in the projection (VIP) value was obtained from each variable in the OPLS-DA model and validation by 7-fold cross-validation and 200 permutation tests. Meanwhile, the non-parametric Mann–Whitney *U* test was conducted to analyze the metabolites, and multiple test corrections using the Benjamini–Hochberg correction were applied to valuate statistical significance. The metabolites with VIP values >1.0 and *Q* value < 0.05 were considered to be statistically significant, whereas variables that were not significantly changed were discarded (32, 33). The metabolites identified were mapped by MS/MS spectral similarity with score ≥0.8 based on an in-house database (34). In addition, the differential metabolites were mapped into their biochemical pathways through metabolic pathway enrichment and pathway analysis based on MetaboAnalyst 5.0 (http://www.metaboanalyst.ca), which uses the high-quality Kyoto Encyclopedia of Genes and Genomes metabolic pathways as the backend knowledge base (34, 35), and a pathway with *p* < 0.05 was considered to be significant (36).

All data analyses were conducted using SPSS 22.0 (IBM Corp., Armonk, NY, USA) or RStudio version 1.2.1335-2009-2019 (RStudio, Inc.) software. Data were assessed for normality of distribution using the Shapiro–Wilk test first. The two-tailed Student *t* test or Mann–Whitney *U* test was used to analyze clinical characteristics (age, BMI, gestational weeks, education level, and EPDS). Heatmap and Spearman's rank correlation analysis of key differential metabolites were conducted using RStudio. Then, the stepwise binary logistic regression analysis and receiver operating characteristic (ROC) curve analysis were performed to explore biomarkers for distinguishing NAD from AD. The area under the curve (AUC) was used to assess the accuracy of diagnostic accuracy, such as 0.8 < AUC < 0.9 as good and 0.9 < AUC ≤ 1.0 as excellent (35). *p* < 0.05 was considered significant.

## Results

### Participant Characteristics

The clinical and anthropometric characteristics of all the participants included in the study are summarized in Table 1. A total of 20 patients with AD and 40 with NAD were recruited for this study. There were no significant differences (*p* > 0.05) in age, BMI of pre-pregnancy and pregnancy, gestational weeks, or education level between the two groups.

**Table 1:** Demographic and clinical characteristics of the antenatal depression and non-antenatal depression.

**Characteristics**	**AD group (*n* = 20)**	**NAD group (*n* = 40)**	***p***
Age in years,^a^ median (IQR)	28.50 (27.00–32.5)	28.5 (27.00–32.5)	0.552
Prepregnancy BMI,^b^ median (IQR)	20.35 (18.75–23.38)	28.5 (27.00–32.5)	0.730
Pregnancy BMI,^b^ median (IQR)	25.76 (24.97–27.55)	28.5 (27.00–32.5)	0.832
Gestational weeks,^b^ median (IQR)	35.00 (34.00–36.00)	28.5 (27.00–32.5)	0.110
University education,^b^ *n* (%)	16 (80%)	37 (92.5%)	0.159
EPDS^b^	12.40 ± 1.60	4.93 ± 2.03	<0.001

*Data presented as mean ± SD, median (IQR), or n (%). AD antenatal depression, NAD non-antenatal depression, IQR interquartile range, BMI body mass index, EPDS Edinburgh Postnatal depression Scale, SD standard deviation*.

a *Analyzed by the Student t test*.

b *Analyzed by the Mann–Whitney U test*.

### LC-MS/MS Metabolomics Analysis

**Figure 1:**
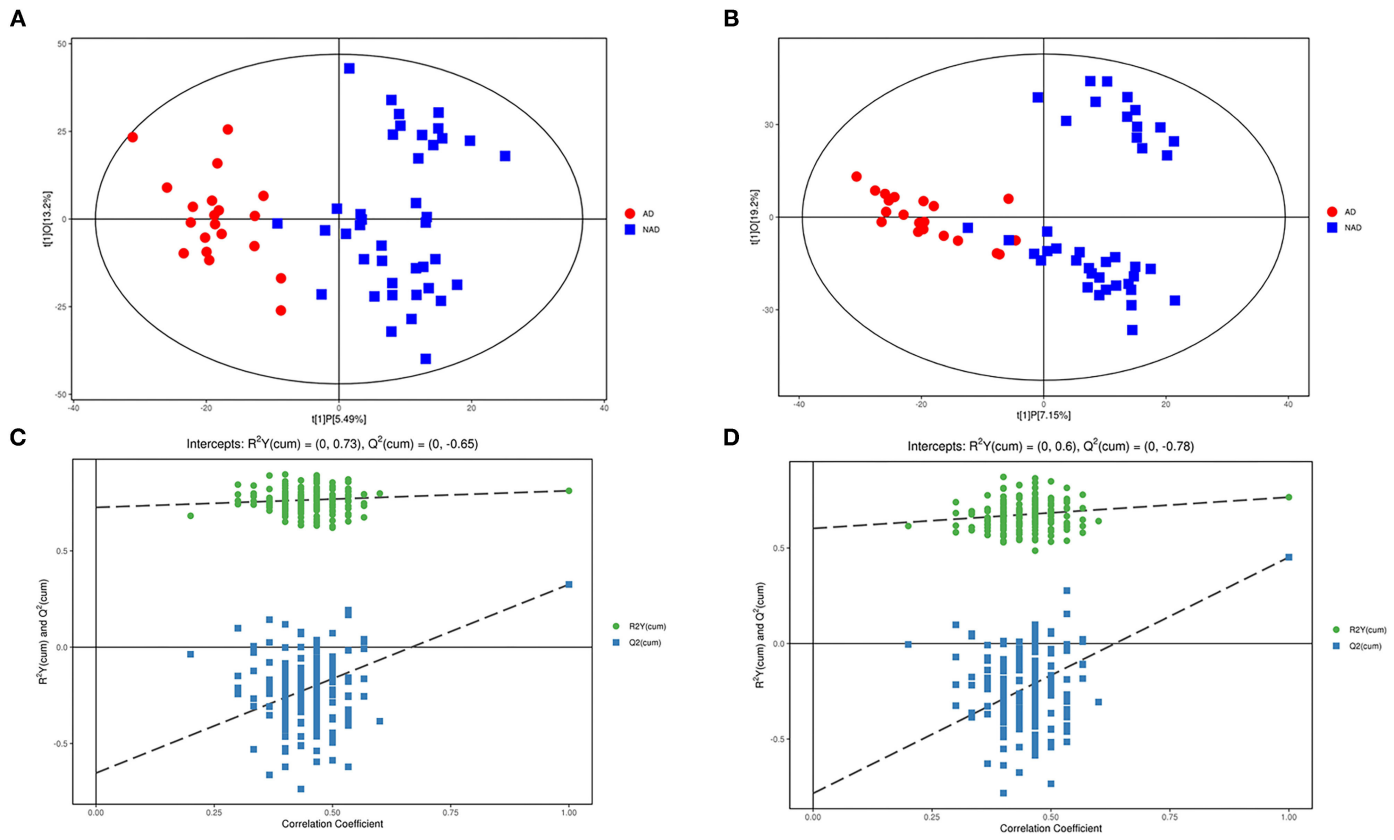
Multivariate statistical analysis. An OPLS-DA model showed that the AD patients (red circle) could be separated from the NAD (blue square) with little overlap in the negative mode **(A)** and positive **(B)**. Validation of the OPLS-DA model by the 200-time permutation test suggested that the original model was valid and not overfitted in negative mode **(C)** and positive **(D)**.

LC-MS/MS metabolomic platform was used to assess the contributions of serum metabolome. After data normalization, the OPLS-DA model revealed that the AD and NAD patients could be significantly separated with little overlap in the negative mode (R2X = 0.187, R2Y = 0.812, Q2 = 0.326, Figure 1A) and positive mode (R2X = 0.264, R2Y = 0. 766, Q2 = 0.452, Figure 1B). Moreover, the 200-permutation test and a typical 7-fold cross-validation were performed, and the results showed that the built model was valid and not over-fitted in the negative mode (R2Y = 0.73, Q2 = −0.65, Figure 1C) and positive (R2Y = 0. 60, Q2 = −0.78, Figure 1D).

### Screening of Differential Metabolites

**Figure 2:**
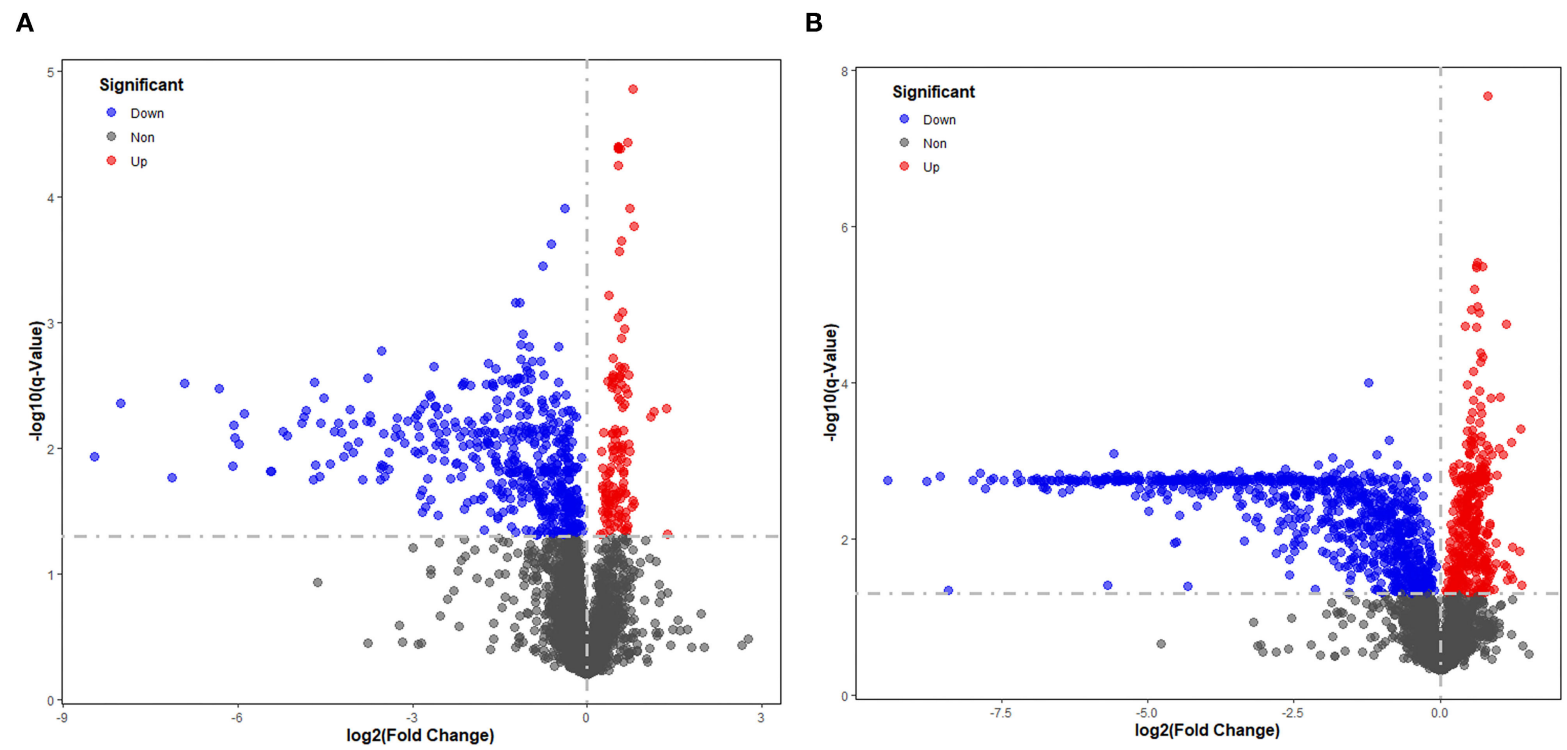
Volcano plot representing the significant variables in the discrimination between AD and NAD groups in the negative mode **(A)** and positive **(B)**. The non-significant and the significant up-regulation and down-regulation variables are represented in gray, red, and blue [*q* < 0.05 and fold change (AD/NAD) >1]. VIP, variable importance in the projection.

Significantly different metabolites were visualized as a volcano plot by plotting the –log10 *q* value (*y* axis) against the corresponding log2 fold change (AD/NAD) (*x* axis), and the differential metabolites were screened according to the set criteria (VIP > 1 and *Q* < 0.05). The volcano plot represents the significant variables in the discrimination between AD and NAD groups in the negative (Figure 2A) and positive (Figure 2B) mode. Then, all the different metabolites were identified and matched by MS/MS spectra based on an in-house database and found that 79 metabolites were significantly different between two groups (Table 2), mainly related to lipid and amino acids.

**Table 2:** Differential metabolites in the serum between the antenatal depression (AD) and antenatal depression (NAD) groups.

**Metabolites name**	**HMDB**	**Class**	**RT**	**VIP**	***Q***	**FC**	**ESI^**+/−**^**
l-Glutamic acid	HMDB0000148	Amino acids	418.1460	1.71	2.85E-03	0.36	–
l-Phenylalanine	HMDB0000159	Amino acids	277.3210	1.65	7.01E-03	0.65	–
l-Serine	HMDB0000187	Amino acids	392.1675	1.05	3.62E-03	0.56	+
N-acetyl-l-phenylalanine	HMDB0000512	Amino acids	209.5230	1.34	1.23E-02	0.65	–
Phenylacetylglycine	HMDB0000821	Amino acids	207.9650	1.28	1.51E-02	0.60	–
l-Norleucine	HMDB0001645	Amino acids	283.8880	1.21	1.17E-02	0.76	–
4-Guanidinobutanoic acid	HMDB0003464	Amino acids	375.5550	1.50	4.25E-03	0.68	+
Arginyl–valine	HMDB0028722	Amino acids	372.8890	1.39	2.68E-02	1.71	+
Asparaginyl–arginine	HMDB0028725	Amino acids	442.1130	1.13	1.13E-02	0.04	+
Glycyl–histidine	HMDB0028843	Amino acids	378.3465	1.48	2.01E-03	0.56	+
Glycyl–valine	HMDB0028854	Amino acids	325.7460	1.66	1.97E-03	0.05	+
Histidinyl–serine	HMDB0028894	Amino acids	381.3160	1.88	3.29E-03	0.14	+
Isoleucyl–leucine	HMDB0028911	Amino acids	212.4180	1.52	1.06E-02	0.10	+
Isoleucyl–phenylalanine	HMDB0028914	Amino acids	201.7700	1.33	2.61E-02	0.59	+
Phenylalanyl–threonine	HMDB0029005	Amino acids	249.9295	1.15	1.45E-02	0.18	+
Ustiloxin D	HMDB0041054	Amino acids	287.7010	1.56	1.82E-03	0.18	+
Apo-[3-methylcrotonoyl-CoA:carbon-dioxide ligase (ADP-forming)]	HMDB0059607	Amino acids	330.6840	1.15	1.84E-02	0.66	+
L-Hexanoylcarnitine	HMDB0000756	Fatty acids	245.3005	1.81	3.74E-02	1.75	+
Valeric acid	HMDB0000892	Fatty acids	101.1280	2.19	8.27E-03	0.02	–
Leukotriene B4	HMDB0001085	Fatty acids	130.4530	1.75	1.76E-02	0.04	–
2-Ethyl-2-hydroxybutyric acid	HMDB0001975	Fatty acids	60.9493	1.03	1.79E-03	0.15	+
Prostaglandin F3a	HMDB0002122	Fatty acids	94.5208	1.57	1.47E-02	0.09	–
Dodecanoylcarnitine	HMDB0002250	Fatty acids	207.0170	1.84	3.04E-02	1.79	+
2-Hydroxymyristic acid	HMDB0002261	Fatty acids	96.1171	1.26	2.46E-02	0.59	–
2,3-Dinor-6-keto-prostaglandin F1 a	HMDB0002277	Fatty acids	93.0783	1.99	7.58E-03	0.26	–
Prostaglandin A2	HMDB0002752	Fatty acids	95.3174	1.72	9.05E-03	0.18	–
9-OxoODE	HMDB0004669	Fatty acids	50.8466	1.31	1.30E-02	0.21	–
Lipoxin B4	HMDB0005082	Fatty acids	76.4873	1.62	1.53E-02	0.02	–
2,6 Dimethylheptanoyl carnitine	HMDB0006320	Fatty acids	218.7390	1.55	1.11E-02	1.51	+
5-HETE	HMDB0011134	Fatty acids	66.1814	1.50	7.89E-03	0.03	–
Aeglin	HMDB0041415	Fatty acids	287.5830	1.62	1.82E-03	0.14	+
Glycerol tripropanoate	HMDB0032857	Complex lipids	62.0970	1.16	1.76E-03	0.19	+
Glycerophosphocholine	HMDB0000086	Complex lipids	403.3310	1.14	6.96E-03	0.44	+
PC (16:0/16:0)	HMDB0000564	Complex lipids	167.0810	2.68	6.33E-06	1.50	+
LysoPC [P-18:1(9Z)]	HMDB0010408	Complex lipids	207.1765	1.17	7.86E-03	0.53	+
2-Ketobutyric acid	HMDB0000005	Compounds	245.6895	1.71	7.64E-03	0.61	–
Betaine	HMDB0000043	Compounds	291.2530	1.25	3.18E-02	1.18	+
Citric acid	HMDB0000094	Compounds	276.0380	1.31	2.58E-03	0.64	+
Choline	HMDB0000097	Compounds	263.6560	1.08	9.27E-03	0.61	+
Hypoxanthine	HMDB0000157	Compounds	182.1340	1.01	2.50E-02	0.67	+
Succinic acid	HMDB0000254	Compounds	411.6870	1.50	1.17E-02	0.80	–
Uridine	HMDB0000296	Compounds	172.8320	1.42	2.22E-02	0.82	–
3-Methyl-2-oxovaleric acid	HMDB0000491	Compounds	41.3037	1.12	3.24E-02	0.77	–
Hydroxypyruvic acid	HMDB0001352	Compounds	417.1300	1.72	1.17E-02	0.76	–
Calcitriol	HMDB0001903	Compounds	33.4882	1.05	1.76E-03	0.27	+
Formylanthranilic acid	HMDB0004089	Compounds	92.6308	1.64	8.80E-03	0.17	–
Nivalenol	HMDB0004304	Compounds	283.5380	1.35	2.52E-03	0.40	+
20-Hydroxyeicosatetraenoic acid	HMDB0005998	Compounds	52.5070	1.20	1.75E-02	0.15	–
Disialosyl galactosyl globoside	HMDB0006588	Compounds	506.8550	1.25	3.27E-03	0.35	+
1-Pyrroline-2-carboxylic acid	HMDB0006875	Compounds	339.6700	1.23	4.04E-03	0.52	+
1-Pyrroline	HMDB0012497	Compounds	330.0500	1.07	2.69E-02	0.87	+
Bortezomib	HMDB0014334	Compounds	287.3350	1.48	3.15E-03	0.25	–
Mitomycin	HMDB0014450	Compounds	189.6270	1.24	1.17E-02	0.56	+
Tamsulosin	HMDB0014844	Compounds	76.5542	1.54	1.75E-03	0.05	+
Fludarabine	HMDB0015206	Compounds	201.1370	1.44	2.28E-02	0.96	+
3-Feruloyl-1,5-quinolactone	HMDB0029289	Compounds	189.9265	1.35	4.78E-03	0.41	+
Ceanothine E	HMDB0029342	Compounds	105.3030	1.53	1.80E-03	0.01	+
1-Methyl-1,3-cyclohexadiene	HMDB0031532	Compounds	31.8345	1.10	1.56E-02	0.76	+
Acrylic acid	HMDB0031647	Compounds	72.0964	1.94	1.45E-02	0.65	–
1,9-Nonanedithiol	HMDB0031710	Compounds	249.6605	1.29	3.97E-02	1.13	+
d-2,3-Dihydroxypropanoic acid	HMDB0031818	Compounds	328.6070	1.86	2.52E-03	0.51	–
3-(1,1-Dimethylallyl)scopoletin 7-glucoside	HMDB0032853	Compounds	286.8130	1.41	1.92E-03	0.26	+
Honyucitrin	HMDB0033536	Compounds	286.8160	1.19	2.00E-03	0.32	+
Ssioriside	HMDB0038934	Compounds	287.6650	1.38	2.33E-03	0.06	+
2-(3,4-Dihydroxyphenylethyl)-6-epi-elenaiate	HMDB0039137	Compounds	285.0410	1.03	2.47E-03	0.33	+
Kanzonol I	HMDB0040606	Compounds	82.8688	1.25	1.73E-03	0.04	+
1-O-2′-Hydroxy-4′-methoxycinnamoyl-b-d-glucose	HMDB0040866	Compounds	190.0090	1.68	2.66E-03	0.50	+
Foeniculoside VII	HMDB0041546	Compounds	71.7108	1.15	1.74E-03	0.13	+
Sofalcone	HMDB0042013	Compounds	287.6165	1.39	1.82E-03	0.24	+
Acetone cyanohydrin	HMDB0060427	Compounds	375.5790	1.50	2.28E-03	0.75	+
N-acetylmuramoyl-ala	HMDB0060494	Compounds	71.9356	1.24	1.84E-03	0.09	+
α-Hydroxytamoxifen	HMDB0060585	Compounds	424.0660	1.70	2.11E-02	1.94	+
Diethyl phthalic acid	HMDB0094660	Compounds	69.2197	1.79	1.78E-02	0.07	–
Cholesterol	HMDB0000067	Steroids	30.9224	1.03	1.58E-03	0.15	+
Cholesta-4,6-dien-3-one	HMDB0002394	Steroids	31.8528	1.49	1.44E-03	0.43	+
Tetrahydroaldosterone-3-glucuronide	HMDB0010357	Steroids	96.0803	1.16	1.74E-03	0.02	+
Betamethasone	HMDB0014586	Steroids	77.2825	1.25	1.75E-03	0.05	+
Fluocinolone acetonide	HMDB0014729	Steroids	82.5630	1.30	1.79E-03	0.03	+
Desglucocheirotoxin	HMDB0034362	Steroids	433.7785	1.71	8.73E-03	1.76	+

a *Metabolites with VIP >1.0 and Q < 0.05 were deemed statistically different. The q value column was based on non-parametric Mann–Whitney U test and Benjamini–Hochberg correction of the normalized MS data*.

b *The FC was calculated by the average mass response (area) ratio (FC = AD/NAD)*.

*ESI, electrospray ionization; VIP, importance in the projection; FC, fold change; Rt, retention time*.

### Functional and Pathway Analysis of Differential Serum Metabolites

**Figure 3:**
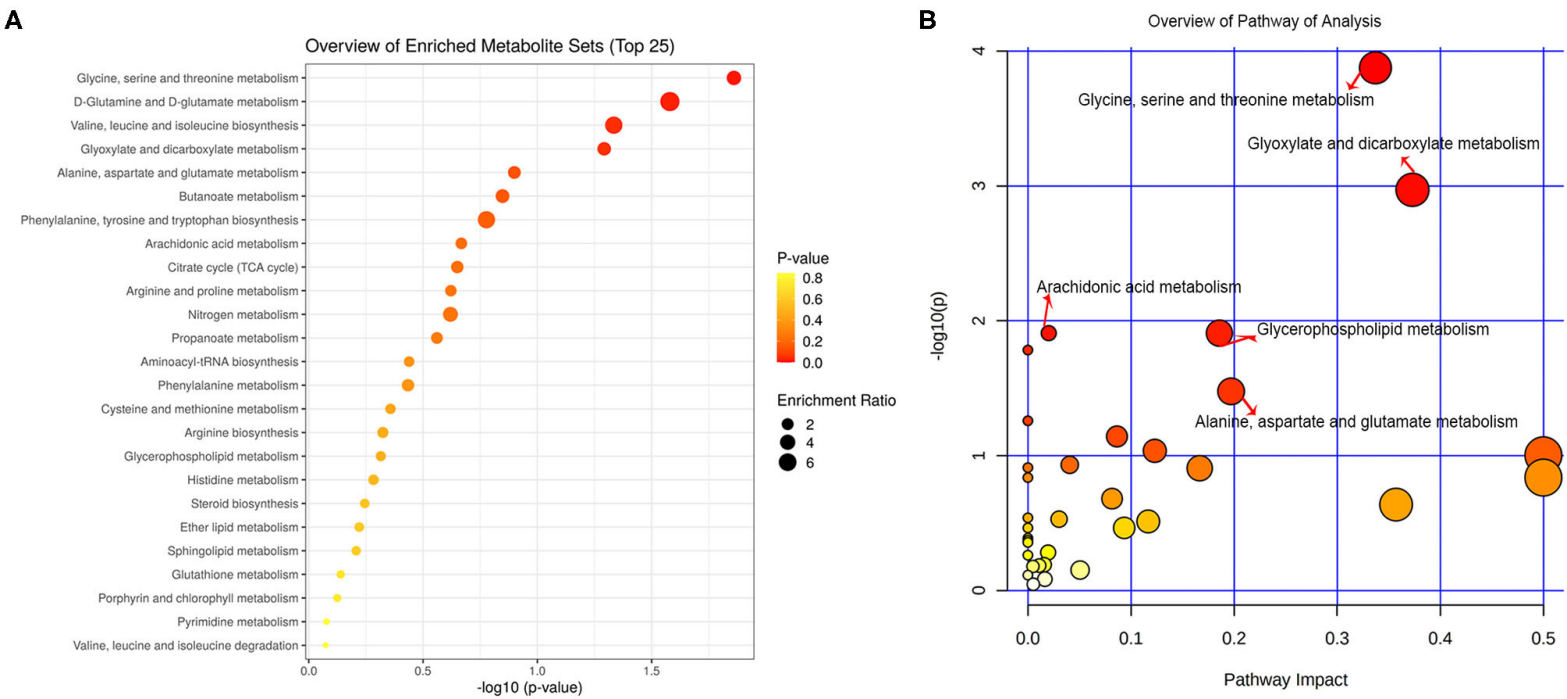
Functional and pathway analysis of differential serum metabolites. Pathway enrichment **(A)** and pathway analysis **(B)** of differential metabolites using MetaboAnalyst 5.0. The *x* axis (pathway impact values) and *y* axis [the –log10 (*p* value)] represent the influencing factor of a topological analysis and the *p* value of the pathway enrichment analysis. The size of the nodes reveals the influence factor of a topological analysis, whereas the color of the nodes indicates the *p* value of the enrichment analysis. The vital metabolic pathways were defined as having *p* < 0.05 and impact value > 0.

All the differential metabolites were mapped into their biochemical pathways through metabolic enrichment and pathway analysis based on the database MetaboAnalyst 5.0, the overview of pathway enrichment and analysis is shown in Figures 3A,B. It revealed evident disorders in five differential metabolic pathways that emerged with pathway impact >0 and *p* < 0.05 (Table 3), including glycerophospholipid metabolism (*p* = 0.0123) and amino acid metabolism, such as glycine, serine, and threonine metabolism (*p* = 0.0001) and Alanine, aspartate, and glutamate metabolism (*p* = 0.0334).

**Table 3:** The top five metabolic pathways analysis via MetaboAnalyst based on all identified differential metabolites.

**No**.	**Pathway name**	**Compound hits**	**p value**	**Pathway impact**	**Metabolites**
1	Glycine, serine, and threonine metabolism	6/33	0.0001	0.34	Choline, betaine, l-serine, hydroxypyruvate, 2-ketobutyric acid, d-2,3-dihydroxypropanoic acid
2	Glyoxylate and dicarboxylate metabolism	5/32	0.0011	0.37	l-Serine, hydroxypyruvate, citric acid, d-2,3-dihydroxypropanoic acid
3	Arachidonic acid metabolism	4/36	0.0123	0.02	20-Hydroxyeicosatetraenoic acid, 5-HETE, leukotriene B4, PC (16:0/16:0)
4	Glycerophospholipid metabolism	4/36	0.0123	0.19	Choline, PC (16:0/16:0), glycerophosphocholine, LysoPC [P-18:1(9Z)]
5	Alanine, aspartate and glutamate metabolism	3/28	0.0334	0.20	Citric acid, l-glutamic acid, succinic acid

**Figure 4:**
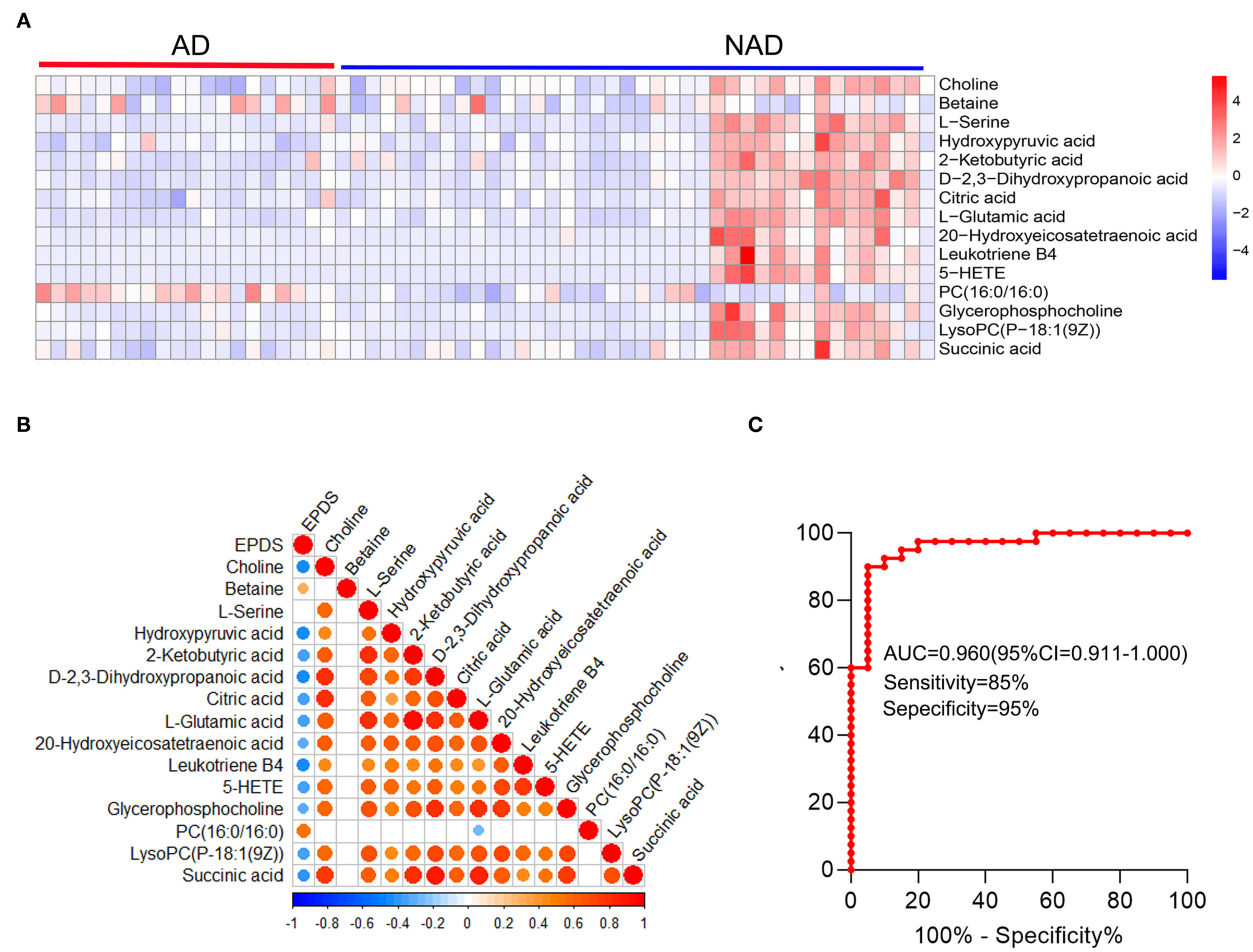
Systems analysis of key differential metabolites. The heatmap visualization of key differential metabolites from the differential pathway **(A)**, Spearman's correlation was conducted between key differential metabolites and EPDS **(B)**, and ROC curve **(C)** of the whole differential metabolites. A significant (*p* < 0.05) positive and negative correlation is indicated with a red and blue color, whereas a non-significant correlation is indicated with blank. EPDS, Edinburgh Postnatal Depression Scale.

Then, the heatmap plotted to visualize the key differential metabolomes, which revealed that the whole metabolome significantly changed in both groups (Figure 4A). The Spearman's correlation was evaluated among the 15 key differential metabolites, and almost all of them are negatively correlated with depression symptom (EPDS), except that PC (16:0/16:0) (*r* = 0.537, *p* < 0.001) and betaine (*r* = 0.329, *p* = 0.010) were significantly positive correlated with EPDS, and l-serine (*r* = 0.198, *p* = 0.129) was not (Figure 4B).

**Figure 5:**
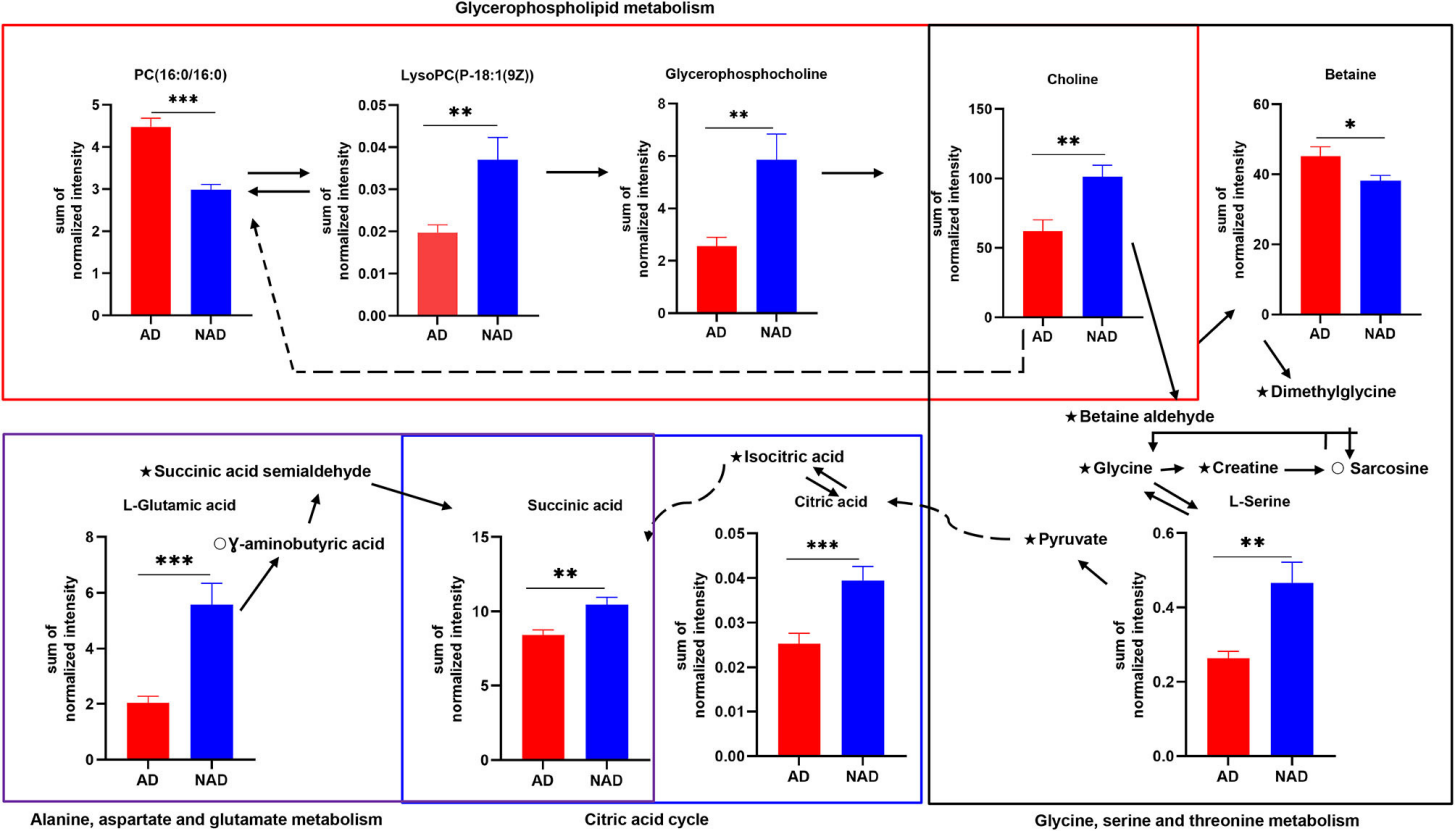
Disturbance of amino acid and glycerophospholipid metabolism in the antenatal depression woman. The key differential metabolites were mainly mapped into the “glycerophospholipid metabolism,” “alanine, aspartate and glutamate metabolism,” and “glycine, serine, and threonine metabolism.” The majority of metabolites of pathways were decreased in the AD group relative to the NAD group. AD, antenatal depression; NAD, non-antenatal depression; PC, phosphatidylcholine; LysoPC, lysophosphatidylcholine. The solid lines show molecular interaction or relation, whereas the dotted lines show indirect or unknown links. Student *t* test; *p < 0.05, **p < 0.01, ***p < 0.001, ⋆, no significance; °, not detected.

The stepwise binary logistic regression analysis of the whole differential metabolites to predicted probability and the ROC analysis to build a curve using the obtained probabilities showed that the identified simplified biomarker panel [betaine, PC (16:0/16:0) and succinic acid] could yield an AUC of 0.960 (95% confidence interval = 0.911–1.000, specificity = 95%, sensitivity = 85%) (Figure 4C). These results demonstrated that this simplified biomarker panel had an excellent diagnostic performance in discriminating AD from NAD. Finally, the molecular interactions related to amino acids and glycerophospholipid metabolism were investigated. The results showed the key metabolites were mainly mapped into the “glycerophospholipid metabolism,” “alanine, aspartate, and glutamate metabolism and glycine,” and “serine and threonine metabolism.” The majority of metabolites of these pathways were decreased in the AD group relative to the NAD group (Figure 5).

## Discussion

To our knowledge, this is the first study to use LC-MS/MS platform to evaluate serum metabolites in late-pregnancy women with and without depression symptoms. In the overall analysis, 79 metabolites were significant different between AD and NAD, which were related to amino acids and lipids (mainly about fatty acid and glycerophosphocholines). Specifically, we found that most of the key metabolites were related to “amino acid metabolism” including “glycine, serine, and threonine metabolism,” “alanine, aspartate and glutamate metabolism,” and “glycerophospholipid metabolism.” Furthermore, correlation analysis revealed that PC (16:0/16:0) and betaine were associated with higher AD risk. Finally, a potential biomarker panel consisting of three serum metabolite biomarkers [betaine, PC (16:0/16:0) and succinic acid] was identified. This panel could discriminate pregnant women with AD from those with NAD with AUC of 0.960.

Depression is a heterogeneous and multifactorial disorder, while the molecular mechanisms that underlie depression remain unclear. Recently, a growing body of evidence suggests that lipid metabolism plays a vital role in the pathological of depression (22, 37–39). Lipids are a fundamental constituent of cellular and subcellular membranes and perform diverse roles in biological functions, such as regulating receptor-mediated signaling processes and transmembrane transport (40). Liu et al. have found that lysophosphatidylcholine (LysoPC), phosphatidylcholine (PC), triacylglycerol (TG), etc., remarkably increased in the plasma of depression patients and showed a significantly positive correlation with depression severity (41). PC is the most abundant phospholipid of all mammalian cell types and subcellular organelles, accounting for 40–50% of total cellular phospholipids (22, 37). LysoPC, an intermediate of PC metabolism, is produced by the cleavage of PC by phospholipase A2 (PLA2) (42), and LysoPC is converted back to PC via lysophosphatidylcholine acyltransferase (43). Then, LysoPC is orderly deacylated to glycerol–phosphocholine and choline by lysophospholipase I and glycerol–phosphocholine phosphodiesterase via glycerophospholipid metabolism pathway (44). In our study, we found that PC (16:0/16:0), choline, LysoPC [P-18:1(9Z)], and glycerol–phosphocholine are related to glycerophospholipid metabolism. PC (16:0/16:0) is up-regulated in the AD compared with those of healthy mothers during pregnancy, whereas LysoPC [P-18:1(9Z)], glycerol–phosphocholine, and choline were down-regulated. The results may be caused by the dysfunction of PC synthesis and metabolism. PC, including PC (16:0/16:0), is primarily made in mammalian cells from choline through the CDP–choline pathway, which accounts for 95% of the total choline pool in mammalian tissue (45), and PC is also metabolized to LysoPC, glycerophospholipid, and choline via glycerophospholipid metabolism pathway (46), in line with previous findings that the levels of PC (16:0/16:0) were obviously increased in the plasma of the AD group (26) and choline and glycerol–phosphocholine were significantly decreased in the urine of patients with moderate depression (Hamilton Depression Scale score ranged from 18 to 24) (47). Masataka et al. suggested that glycerol–phosphocholine can prevent the aging-related decline in cognitive function, which plays a vital role in sustaining structural and functional integrity of cellular membranes including neuronal membranes (48). In addition, Zheng et al. have also found that the metabolites in hippocampal depressive macaques were significantly different from those of controls, which were mainly enriched in glycerophospholipid metabolism (49). Given that the changes of PC (16:0/16:0), choline, LysoPC [P-18:1(9Z)], and glycerol–phosphocholine are inconsistent, alterations of any of them would cause disturbance of glycerophospholipid metabolism. Thus, the disturbance of glycerophospholipid metabolism is associated with depression.

Depression, a serious mental illness, is influenced by genes and the environment. It has reported that the environmental factors regulated the gene expression and function of neuronal membrane via epigenetic mechanisms (50). Choline is a necessary nutrient and acts as an indirect methyl donor, which is required for normal brain growth and development (51). Choline was also a precursor of the neurotransmitter acetylcholine, PC, and betaine, involved in several critical physiological functions (52), such as modulation of gene expression, synaptic plasticity, and cholinergic signaling (50, 51). Betaine is a direct methyl donor, which can be obtained from diet or transformed from choline (53). A recent study revealed that the concentrations of choline and betaine were significantly positive and a trending positive correlation with depression scores (EPDS), respectively (54), and betaine was associated with the severity of depression of psychiatric patients, who were diagnosed with major depressive disorder (MDD) or bipolar disorder (55). Thus, the levels of choline and betaine are associated with the clinical status of depression. In the present study, the choline was an intermediate between disturbance of glycerophospholipid metabolism and glycine, serine, and threonine metabolism, and the choline is distinctly lower in AD women than the controls; betaine is observed to be increased in the AD group and involved in disturbance of glycine, serine, and threonine metabolism. In line with previous findings, the levels of betaine increased in urine samples from subjects with bipolar disorders; choline was down-regulated in the plasma of depressed patients and reversed to the normal levels after Xiaoyaosan treatment (56). The results may explain the oxidization of choline into betaine (57). In addition, total choline and betaine significantly decreased in the AD group, compared with those from the NAD group. Both choline and betaine are involved in one-carbon metabolism, including folate and methionine cycles (58). Choline can indirectly donate its methyl groups and participate in folate-mediated one-carbon metabolism via its oxidation to betaine by choline oxidase (50), while betaine is an essential methyl donor for the methionine–homocysteine cycle. Based on the above evidence, the change in choline and betaine disturbs the one-carbon metabolism and influences the expression of pivotal genes via epigenetic mechanisms, and these genes were related to memory, learning, and cognitive functions.

Accumulating evidence supports that amino acid metabolism and glutamatergic system are involved in the pathogenesis of depression (21, 59). A vast evidence proved that the glutamatergic system can be a novel therapeutic target of MDD, particularly via *N*-methyl-d-aspartate receptors (NMDARs) (60), such as NMDAR antagonist ketamine (61). In this study, we found that l-glutamic acid (Glu), succinic acid, and citric acid decreased in the serum of pregnant women with depression, which was related to alanine, aspartate, and glutamate metabolism pathway. In line with previous studies, Glu decreased in the dorsolateral prefrontal cortex of individuals with depression, as well as in the anterior cingulate cortex (62). In addition, succinate and citrate are important for the tricarboxylic acid (TCA) cycle. TCA cycle is the final common oxidative pathway and most effective energy metabolism pathway. Succinate is an important intermediate of the TCA cycle, and it also interacts with the metabolism of the Glu–γ-aminobutyric acid–glutamine pathway (63). Glu also can be a substrate for the TCA cycle, as it can be converted to α-ketoglutarate by transaminases or glutamate dehydrogenase (64). It is the main excitatory neurotransmitter released by synapses in the central nervous system and regulates synaptic plasticity, cognitive processes, and reward and emotional processes (65). Therefore, Glu signaling is at the crossroad of multiple metabolic pathways and, accordingly, including the influence of TCA cycle and the “alanine, aspartate, and glutamate metabolism” pathway. The dysfunction of TCA may play a role in the pathophysiology of depression.

## Limitations

Some limitations need to be addressed. First, relatively small sample size and late-pregnancy subjects were enrolled in this study; future studies with large-scale samples are still needed to validate our study. Second, all subjects were from the same location and might share the same dietary habits, which may restrict the generalization of the findings. Third, only serum metabolites were studied, and further studies should collect other biological samples from the same subjects. Fourth, the number of differential metabolites might be not enough to obtain the robust results of pathway analysis and enrichment analysis; thus, future studies are still needed to validate and support these results. Therefore, these findings shed new light to further elucidate the molecular mechanism of depression. And our preliminary investigation found that studies on potential biomarkers for AD and key metabolic pathways are needed for further validation.

## Conclusion

In summary, 79 significant differential metabolites between AD and NAD were identified by LC-MS/MS. We also found that these metabolites mainly influenced “amino acids metabolism” and “glycerophospholipid metabolism.” Meanwhile, potential serum diagnostic metabolite panels [betaine, PC (16:0/16:0) and succinic acid] clearly discriminated AD from NAD with excellent accuracy. These findings may aid in uncovering the molecular pathogenesis of AD and then prompting the development of diagnostic and prognostic tests for the disorder.

## Data Availability Statement

The original contributions presented in the study are included in the article/Supplementary Material, further inquiries can be directed to the corresponding authors.

## Ethics Statement

The studies involving human participants were reviewed and approved by the Medical Ethics Committee of the affiliated hospital, Guizhou Medical University. Written informed consent to participate in this study was provided by the participants' legal guardian/next of kin.

## Author Contributions

TZ: study concept and design. TT, XG, and XZ: performed the experiments. TT: experimental technical guidance. QM: data analysis, manuscripts drafting, and revised. All authors reviewed and approved the manuscript before its submission.

## Conflict of Interest

The authors declare that the research was conducted in the absence of any commercial or financial relationships that could be construed as a potential conflict of interest.
